# Shared and Disorder-Specific Neurocomputational Mechanisms of Decision-Making in Autism Spectrum Disorder and Obsessive-Compulsive Disorder

**DOI:** 10.1093/cercor/bhx265

**Published:** 2017-10-17

**Authors:** Christina O Carlisi, Luke Norman, Clodagh M Murphy, Anastasia Christakou, Kaylita Chantiluke, Vincent Giampietro, Andrew Simmons, Michael Brammer, Declan G Murphy, David Mataix-Cols, Katya Rubia, MRC AIMS consortium

**Affiliations:** 1 Department of Child and Adolescent Psychiatry, Institute of Psychiatry, Psychology and Neuroscience, King's College, London, UK; 2 Department of Forensic and Neurodevelopmental Sciences, Sackler Institute for Translational Neurodevelopmental Sciences, Institute of Psychiatry, Psychology and Neuroscience, King's College, London, UK; 3 Behavioural Genetics Clinic, Adult Autism Service, Behavioural and Developmental Psychiatry Clinical Academic Group, South London and Maudsley Foundation NHS Trust, UK; 4 Centre for Integrative Neuroscience and Neurodynamics, School of Psychology and Clinical Language Sciences, University of Reading, Reading, UK; 5 Department of Neuroimaging, Institute of Psychiatry, Psychology and Neuroscience, King's College, London, UK; 6 National Institute for Health Research (NIHR) Biomedical Research Centre (BRC) for Mental Health at South London and Maudsley NHS Foundation Trust and Institute of Psychiatry, Psychology and Neuroscience, King's College London, London, UK; 7 Department of Neurobiology, Care Sciences and Society, Center for Alzheimer Research, Division of Clinical Geriatrics, Karolinska Institutet, Stockholm, Sweden; 8 Department of Clinical Neuroscience, Centre for Psychiatry Research, Karolinska Institutet, Stockholm, Sweden

**Keywords:** Autism Spectrum Disorder, computational modeling, decision-making, fMRI, obsessive-compulsive disorder

## Abstract

Autism spectrum disorder (ASD) and obsessive-compulsive disorder (OCD) often share phenotypes of repetitive behaviors, possibly underpinned by abnormal decision-making. To compare neural correlates underlying decision-making between these disorders, brain activation of boys with ASD (*N* = 24), OCD (*N* = 20) and typically developing controls (*N* = 20) during gambling was compared, and computational modeling compared performance. Patients were unimpaired on number of risky decisions, but modeling showed that both patient groups had lower choice consistency and relied less on reinforcement learning compared to controls. ASD individuals had disorder-specific choice perseverance abnormalities compared to OCD individuals. Neurofunctionally, ASD and OCD boys shared dorsolateral/inferior frontal underactivation compared to controls during decision-making. During outcome anticipation, patients shared underactivation compared to controls in lateral inferior/orbitofrontal cortex and ventral striatum. During reward receipt, ASD boys had disorder-specific enhanced activation in inferior frontal/insular regions relative to OCD boys and controls. Results showed that ASD and OCD individuals shared decision-making strategies that differed from controls to achieve comparable performance to controls. Patients showed shared abnormalities in lateral-(orbito)fronto-striatal reward circuitry, but ASD boys had disorder-specific lateral inferior frontal/insular overactivation, suggesting that shared and disorder-specific mechanisms underpin decision-making in these disorders. Findings provide evidence for shared neurobiological substrates that could serve as possible future biomarkers.

## Introduction

Autism Spectrum Disorder (ASD) is characterized by social and communication difficulties and restricted, repetitive behaviors (American Psychiatric Association 2013) and affects 0.6–2.0% of the population, with a higher prevalence in males (Blumberg et al. 2013). Obsessive-Compulsive Disorder (OCD) is identified by recurrent and intrusive distressing thoughts (obsessions) and repetitive rituals (compulsions) (American Psychiatric Association 2013) and has a prevalence of 1–3%, with a slightly higher incidence in males in pediatric samples (Ruscio et al. 2010). These highly heterogeneous and frequently comorbid disorders can sometimes be clinically difficult to separate, as symptoms such as repetitive behaviors in ASD can often resemble OCD-related compulsions (Russell et al. 2005). Such overlap has been attributed to shared genetic risk and biological mechanisms as well as diagnostic mislabelling (Russell et al. 2016), highlighting a need to understand the distinct and overlapping underlying neurobiological mechanisms of both disorders.

Executive functions (EF) are higher-order cognitive functions important for goal-directed behavior and can be conceptualized dichotomously as “cool” EF, referring to nonemotional functions including inhibition and working memory, and “hot” EF, referring to functions with reward-based motivation including gambling and reward learning (Zelazo and Müller 2007). Cool EF has been widely investigated in ASD and OCD (for reviews, see (Zelazo and Müller 2007; van Velzen et al. 2014; Carlisi, Norman, Lukito et al. 2016; Norman et al. 2016)). However, relatively less is known about the mechanisms underlying reward-related hot EF processes in these disorders, as evidence to date has been inconsistent.

Impaired decision-making has been implicated in both ASD and OCD (Cavedini et al. 2006; Luke et al. 2012). The Iowa Gambling Task (IGT) (Bechara et al. 1994) has been widely used in typically developing populations to measure reward-based decision-making and temporal foresight impairments under conditions of ambiguity, as it requires reinforcement learning to distinguish between choices that yield large immediate gains but even larger losses (risky options) leading to long-term financial losses and decks that give small gains but even smaller losses, leading to long-term financial gains at the end of the game (safe options).

There have been only 5 studies in ASD using the IGT (Johnson et al. 2006; Yechiam et al. 2010; South et al. 2014; Mussey et al. 2015; Zhang et al. 2015), showing mixed results. A relatively consistent finding in both children/adolescents (Johnson et al. 2006; Yechiam et al. 2010) and adults (Mussey et al. 2015) is that ASD individuals shift more frequently between choices, possibly due to difficulties with implicit learning (Johnson et al. 2006) or exploration-focused learning strategies (Yechiam et al. 2010). Another study in adults with ASD found that the ASD group had worse performance, preferring disadvantageous decks (Zhang et al. 2015). However, one study (South et al. 2014) in children/adolescents found superior performance in ASD adolescents relative to typically developing controls, explained by a “loss-avoidance” style of decision-making in the ASD group in contrast to a “reward-seeking” style often observed among typically developing adolescents (Smith et al. 2012).

There have been relatively more studies using the IGT in adults with OCD (e.g., (Purcell et al. 1998; Cavedini et al. 2002, 2010; Cavallaro et al. 2003; Olley et al. 2007; Starcke et al. 2010; Rocha et al. 2011; Grassi et al. 2015; Kim et al. 2015)). The majority show impaired decision-making in patients relative to controls, with patients preferring large immediate rewards and not learning from losses, although there have also been negative findings (Nielen et al. 2002; Lawrence et al. 2006; Krishna et al. 2011). Only one study was conducted in children with OCD using the IGT which found that patients performed worse relative to controls and that this was related to symptom severity during the most severe period of illness (Kodaira et al. 2012).

The IGT taps a range of cognitive processes including reward-related decision-making, reward sensitivity, loss aversion, temporal foresight, inhibitory control (to inhibit the contextual “thrill” of immediate gains), and exploratory behavior. Thus, to clarify IGT performance impairments (or lack thereof) in both clinical groups, it is important to investigate these cognitive and motivational factors on a more nuanced level to better characterize task performance, and computational modeling is a useful tool for this (Huys et al. 2016).

Similar performance deficits could also be mediated by different underlying neurofunctional networks. No functional magnetic resonance imaging (fMRI) studies, however, have yet investigated the neural correlates of decision-making under ambiguity in ASD or OCD using the IGT. In typically developing individuals, the IGT activates dorsolateral and ventromedial prefrontal, orbitofrontal, insular, posterior cingulate, and ventral striatal regions during the various stages of the decision-making process (Li et al. 2010). In light of a dearth of evidence in ASD and OCD specifically on the IGT, evidence can be compiled from studies examining related reward-based decision-making processes; during tasks of temporal discounting (Chantiluke, Christakou et al. 2014) and reversal-learning (Chantiluke, Barrett, Giampietro, Brammer et al. 2015), adolescents with ASD have shown abnormalities in related fronto-temporo-limbic systems mediating executive processes (Carlisi, Norman, Lukito et al. 2016) and ventromedial/fronto-limbic regions important for reward-related functions, especially those involving monetary gain/loss (Kohls et al. 2013). OCD has traditionally been conceptualized as a disorder of abnormalities in ventral affective systems including (orbito)fronto-striato-thalamo-cortical networks as well as in lateral orbitofrontal-striatal systems important for cognitive/inhibitory control (Zelazo and Müller 2007; Menzies et al. 2008; Carlisi, Norman, Lukito et al. 2016). fMRI studies involving reward-related decision-making support evidence for abnormalities in both motivation control as well as cognitive control regions by showing that OCD patients relative to controls have hyperactivity in ventromedial prefrontal, orbitofrontal and anterior cingulate cortex (ACC) regions projecting to ventral striatum and medio-dorsal thalamus, and underactivation in cortico-striato-thalamic regions including dorsolateral prefrontal cortex (DLPFC), temporal and parietal cortices, and basal ganglia (Menzies et al. 2008; Brem et al. 2012).

The relative lack of consistent findings in ASD and OCD on the IGT highlights a need for a better understanding of neurocognitive phenotypes of reward-based decision-making in these disorders. Recent efforts such as the Research Domain Criteria (RDoC; (Insel et al. 2010)) stress the importance of investigating trans-diagnostic phenotypes which may be underpinned by shared and/or disorder-specific neurofunctional mechanisms. Thus, we compared adolescents with ASD to those with OCD and typically developing controls to investigate shared and disorder-specific brain function abnormalities during the IGT and compared reinforcement learning models to examine fine-grained differences in behavioral factors that might underlie overall decision-making. We hypothesized that both patient groups would be impaired on some aspect of task performance. Specifically, we hypothesized that OCD adolescents would show increased risky decision-making on the IGT compared to typically developing controls as evidenced by previous studies (Starcke et al. 2010; Grassi et al. 2015). Moreover, we hypothesized that OCD boys would show more brain-based impairments during loss and negative outcome based on the literature in this patient group of impaired error monitoring (Fitzgerald et al. 2005) and the clinical literature of the prototypical feeling that things need to be “just right” which often characterizes individuals with OCD (Coles et al. 2003). For ASD boys, we hypothesized this group would show lower choice consistency compared to typically developing control participants (Johnson et al. 2006; Yechiam et al. 2010) and OCD patients. We tested whether differences were due to more nuanced shared or disorder-specific differences in decision-making styles. Based on evidence from IGT studies in typically developing individuals showing that reward-based decision-making may be driven by dorsolateral and ventromedial/orbitofronto-striato-limbic function (Li et al. 2010; Christakou, Gershman et al. 2013), we hypothesized that both groups would show abnormalities in these networks (Christakou et al. 2011; Brem et al. 2012). Furthermore, based on prior evidence of neurofunctional reward-related deficits in the 2 disorders, we hypothesized that both disorders would show abnormal reward processing in ventromedial-fronto-temporo-limbic (Kohls et al. 2013) regions important for reward-based decision-making and temporal foresight required by the task (Menzies et al. 2008). However, we also expected disorder-specific stronger deficits in OCD in orbitofrontal regions and in ASD in ventral striatal and anterior cingulate regions based on respective deficits in these regions observed in each disorder (Menzies et al. 2008; Kohls et al. 2013).

## Materials and Methods

### Participants

64 right-handed (Oldfield 1971) boys (20 typically developing control boys, 24 boys with ASD, 20 boys with OCD), 11–17 years-old, IQ ≥ 70 (Wechsler 1999) participated. Medication-naïve ASD boys were recruited from local clinics. Clinical ASD diagnosis was made by a consultant psychiatrist using ICD-10 research diagnostic criteria (WHO 1992) and confirmed using the Autism Diagnostic Interview-Revised (ADI-R (Lord et al. 1994)). The Autism Diagnostic Observation Schedule (ADOS (Lord et al. 1989)) was also completed. All ASD boys reached clinical thresholds in all domains on the ADI-R (social, communication, restricted/stereotyped behavior) and ADOS (communication, social). Parents of ASD boys also completed the Social Communication Questionnaire (SCQ; (Rutter et al. 2003)) and the Strengths and Difficulties Questionnaire (SDQ; (Goodman and Scott 1999)). ASD participants had a physical examination to exclude comorbid medical disorders and any abnormalities associated with ASD. Individuals with comorbid psychiatric conditions, including OCD and ADHD, were not included.

OCD boys were recruited from the Maudsley Hospital National & Specialist OCD clinic. Diagnosis was made by a consultant clinician using ICD-10 criteria and confirmed with the Children's Yale-Brown Obsessive-Compulsive Scale (CY-BOCS; (Goodman et al. 1989)) and ancillary symptom checklist. Parents of OCD boys also completed the SDQ. OCD patients with comorbid psychiatric or neurological conditions, including ASD and ADHD, were excluded. Four boys were prescribed stable doses of antidepressants (see Supplement).

Twenty age- and handedness-matched typically developing control boys were recruited locally by advertisement. Controls did not meet clinical thresholds on the SDQ and SCQ for any disorder and did not have a current or lifetime history of any psychiatric condition.

Exclusion criteria for all subjects were comorbid psychiatric/medical disorders affecting brain development (e.g., epilepsy/psychosis), drug/alcohol dependency, history of head injury, genetic conditions associated with autism, abnormal structural MRI scans, and MRI contraindications. Controls also participated in our fMRI study examining maturation of decision-making on the IGT, published previously (Christakou, Gershman et al. 2013). Most ASD and control participants also participated in additional fMRI tasks during their visit, published elsewhere (Christakou et al. 2011; Christakou, Murphy et al. 2013; Chantiluke, Barrett et al. 2014; Murphy et al. 2014; Chantiluke, Barrett, Giampietro, Brammer et al. 2015; Chantiluke, Barrett, Giampietro, Santosh et al. 2015; Carlisi, Chantiluke et al. 2016).

This study was conducted in accordance with the Declaration of Helsinki. Ethical approval was obtained from the local Research Ethics Committee (05/Q0706/275). Study details were explained to participants and guardians. Written, informed assent/consent was obtained for all participants, and individuals were compensated for their time and travel expenses.

### Iowa Gambling Task

The fMRI version of the IGT used in this study is described in detail elsewhere (Christakou et al. 2009; Christakou, Gershman et al. 2013). Briefly, on each of 80 trials, participants were presented with 4 card decks (A/B/C/D) on a screen and instructed to choose any deck by pressing the corresponding button with the right hand on an MR-compatible 5-button response box. They were instructed to win as much money as possible by the end of the task. They were only told that sometimes they would win money and sometimes they would lose money, and that some decks might be better than others. They were also told that their final amount won on the task would determine how much of a maximum £30 they would receive as compensation (in reality, all subjects received £30).

Decks A and B were termed disadvantageous or “risky” decks because they returned relatively large gains (£190/£200/£210) but even larger losses (£240/£250/£260), leading to an overall net loss, whereas decks C and D were advantageous or “safe” because they returned small gains (£90/£100/£110) but even smaller losses (£40/£50/£60), resulting in a net gain. There was a 50% probability of winning or losing on each deck.

Task performance is summarized by the ratio of advantageous choices to total choices or, the number of cards picked from decks C + D divided by the total number of cards picked (A + B + C + D). This ratio is proportional to the “net score” ((C + D) − (A + B)) frequently used when quantifying performance on the IGT (Bechara et al. 1994) without giving negative values. Ratios above 0.5 denote preference for safe relative to risky decks, while a ratio below 0.5 implies perseveration on risky choices despite accumulating losses. Responses where reaction time (RT) was less than 200 ms were considered “premature” and these trials were not included in analyses (Thorpe et al. 1996).

**Figure 1.**
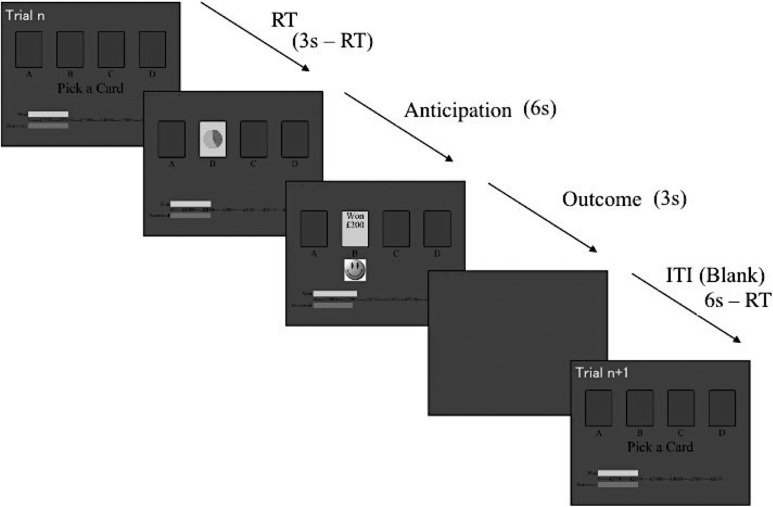
Schematic diagram of the IGT (Christakou, Gershman et al. 2013) Participants were initially “loaned” £2000, indicated by the red bar, and money won/lost was added to/deducted from this amount, indicated by the current running total, depicted by the green bar. At the start of each trial, participants were presented with 4 decks of cards and asked to choose one by pressing with the right hand one of 4 buttons on an MR-compatible response box. Participants were given 3 s to make a response, and their choice (reaction time – RT) was followed by an anticipation period of 6 s, during which a 12-segment circle was presented that counted down the 6 s in steps of 0.5 s. Outcome of the decision (wins = green card, happy face; losses = red card, sad face) was presented for 3 s, after which a blank screen (inter-trial interval – ITI) was presented for a variable 3 s, determined by the RT, resulting in a total trial duration of 15 s (RT (up to 3 s) + anticipation (6 s) + outcome (3 s) + ITI (3 s or more if RT was less than 3 s) = 15 s).

This IGT adaptation differs from other fMRI versions (e.g., (Lawrence et al. 2009)) in that choice was temporally separated from its outcome, haemodynamically decoupling choice and outcome evaluation, allowing separate examination of each. Subjects were given 3 s to respond. Following each choice, the chosen deck was superimposed with a 12-segment wheel ticking down every 0.5 s for a total 6 s until outcome presentation. If no response was made, the trial progressed directly to a blank screen for 9 s. Positive (win) and negative (loss) outcomes were indicated by a happy or sad face presented below the deck and the amount won or lost indicated on the card (Fig. 1). Outcomes were presented for 3 s. Trials lasted 15 s, ending with a blank screen after outcome presentation serving as an implicit baseline in the fMRI analysis. Omitted trials were excluded from analyses. The length of each inter-trial interval (ITI) was determined by the RT, which jittered trial events so as to maintain a 15 s total trial duration. As these manipulations lengthened trial and task duration compared to other behavioral variants, this version of the task included 80 trials rather than the typical 100 trials (Bechara et al. 1994; Lawrence et al. 2009). Total task time was 21 min. Before testing, participants practiced the task in a mock scanner, where 10 test trials presented equal payoffs across decks.

### Computational Modeling

The IGT requires decision-making based on the learned outcomes of previous choices. Performance on the IGT can be influenced by a range of factors including learning rates, reward and loss sensitivity, or inconsistent responding (Ahn et al. 2014). Thus, computational approaches are especially useful for understanding the processes underlying IGT performance. We used hierarchical Bayesian analysis (HBA) implemented within the *hBayesDM* R package (https://cran.r-project.org/web/packages/hBayesDM/index.html) for computational modeling of IGT performance (Ahn et al. 2016). For further details of the methods, rationale and advantages of HBA over other modeling methods (e.g., maximum likelihood estimation), see Supplement and (Lee 2011). HBA involves preparation of trial-by-trial task data for each participant, model fitting and comparison of 3 commonly used and validated models of the IGT: the “Prospect Valence Learning (PVL)-Decay Reinforcement Learning (RI) model, the PVL-Delta model and the Value-Plus-Perseverance (VPP)“ model (Worthy, Pang et al. 2013; Ahn et al. 2014; Steingroever et al. 2014).

The PVL models focus on 4 parameters based on learning theory: α represents feedback sensitivity, λ represents loss aversion, *c* represents choice consistency, and *A* represents learning rate (how much weight is placed on past experiences of a chosen deck vs. the most recent experience of that deck). These models are identical except that they use different learning rules; in the PVL-decayRI rule, expectancies of all decks are discounted on each trial, but in the PVL-Delta rule, only the expectancy of the selected deck is updated.

Based on previous simulation experiments (Worthy, Hawthorne et al. 2013), the VPP model combines the learning rule of the PVL-Delta model with the perseverance heuristic of win-stay-lose-switch choice behavior. This model contains 4 additional perseverance parameters: *k* determines how much the perseverance strengths of all decks decay on each trial, ε_p_ and ε_n_ indicate loss/gain impact, respectively, on choice behavior (i.e., stay/switch tendency), and ω is the reinforcement learning weight, that is, the degree on which a subject relies on reinforcement learning over perseverative strategies. For complete model details, see Supplementary and (Ahn et al. 2014, 2016).

### Model Fitting and Comparison

Posterior inference for all models was performed via Markov Chain Monte Carlo (MCMC) sampling implemented in RStan (http://mc-stan.org/interfaces/rstan). Stan (v2.1.0 (Carpenter et al. 2017)) uses a specific probabilistic sampler called Hamiltonian Monte Carlo (HMC) to sample from the posterior distribution. For details, see (Kruschke 2014; Ahn et al. 2016) and the Stan reference manual (http://mc-stan.org/documentation/).

*hBayesDM* enables model-fit assessment and post hoc comparison via Widely Applicable Information Criterion (WAIC) (Watanabe 2010). This index is obtained by computing the summed point-wise log-likelihood per participant, accounting for the fact that in the IGT, choices on a given trial are dependent on previous choices (Gelman et al. 2014). Smaller WAIC scores denote better model-fit, and overall fit is assessed by adding WAIC scores from each group for each model.

### Statistical Analysis

All analyses were conducted in JASP (v0.8.1.1;https://jasp-stats.org/) using Bayesian analysis based on posterior probabilities rather than frequentist *p*-values, which rely on the sampling intentions of the investigator. Models were favored if BF_10_ > 10, indicating strong evidence for the tested model over the null hypothesis. In instances where BF_10_ was sufficiently large (>1000), Log(BF_10_) is reported, where values >1 indicate strong evidence for the model. For clarity, where appropriate, we also report null hypothesis significance test (NHST) results, including *P*-values.

ANOVAs tested for group differences in demographic and questionnaire measures, and in task performance. Group differences in mean parameter estimates were assessed by each parameter's highest density interval (HDI), i.e., the range of parameter values that spans 95% of the distribution in a pairwise comparison (Ahn et al. 2014). A parameter was considered to significantly differ between groups if the HDI did not overlap 0. Nonparametric correlations (Kendall's Tau rank coefficients) were conducted to test for associations between task performance, symptoms and brain activation.

### fMRI Acquisition

Gradient echo echo-planar magnetic resonance imaging data were acquired on a GE Signa 3-Tesla scanner (General Electric, Waukesha WI) at the Centre for Neuroimaging Sciences, King's College London, using a semi-automated image quality-control procedure (Simmons et al. 1999). A quadrature birdcage head coil was used for radiofrequency transmission and reception. In each of 22 noncontiguous planes, we acquired 800 T_2_*-weighted images depicting blood oxygenation-level dependent (BOLD) response covering the whole-brain (echo time (TE) = 30 ms, repetition time (TR) = 1.5 s, flip angle=60°, in-plane resolution = 3.75 mm, slice thickness = 5.0 mm, slice skip = 0.5 mm). A whole-brain high-resolution structural image with 43 slices was also acquired (TE = 40 ms, TR = 3 s, flip angle=90°, slice thickness, 3.0 mm, slice skip = 0.3 mm).

### fMRI Data Analysis

fMRI data were analysed using a nonparametric permutation-based software developed at the Institute of Psychiatry, Psychology and Neuroscience (XBAM v4.1; http://brainmap.co.uk) which avoids issues such as false positives that are related to parametric statistical analyses (Eklund et al. 2016). In contrast to normal theory-based inference, this approach minimizes assumptions and uses median rather than mean-based statistics to control for outlier effects. Its most commonly used test statistic is computed by standardizing for individual differences in residual noise before performing second-level multi-subject testing using robust permutation-based methods. This allows a mixed-effects approach to analysis that has been recommended following analysis of the validity and impact of theory-based inference in fMRI (Thirion et al. 2007). Details of individual and group-level analyses are described elsewhere (Christakou et al. 2009) and in the Supplement.

Briefly, fMRI data were realigned to minimize motion-related artefacts and smoothed with a Gaussian filter (full-width at half-maximum 8.82 mm) (Bullmore et al. 1999). Time-series analysis of individual subject activation was performed with wavelet-based resampling described in (Bullmore et al. 2001). We first convolved the task epoch of each event of interest (choice, anticipation, outcome) with 2 Poisson model functions (4 and 8 s delays). Using rigid-body and affine transformation, individual maps were registered into Talairach space (Talairach and Tournoux 1988). Group maps were then produced for each experimental condition, and hypothesis testing was performed using cluster-level analysis, shown to give excellent cluster-wise type-I error control (Bullmore et al. 2001). Time-series permutation was used to compute the distribution of the statistic of interest under the null hypothesis. The voxel-level threshold was set to 0.05 to give maximum sensitivity and to avoid type-II errors. Then, a cluster-mass threshold was computed from the distribution of cluster masses in the wavelet-permuted data such that the final expected number of Type-I error clusters under the null hypothesis was less than one per whole-brain. Given that brain activation changes with age during development (Rubia et al. 2010, 2013), and hence to control for possible effects of nonsignificant group differences in age, age was included as a covariate of no interest in the fMRI analyses. However, because groups did not differ in age, analyses were repeated to confirm that inclusion of this covariate did not significantly affect results.

To more specifically focus on areas implicated in the IGT and reward/punishment processing (Li et al. 2010), additional analyses were conducted using a region of interest (ROI) approach based on a priori hypotheses. Search space was restricted to a single mask comprising bilateral orbitofrontal cortex, medial frontal gyrus, inferior frontal gyrus (opercularis), inferior frontal gyrus (triangularis), insula, putamen, caudate, and nucleus accumbens. Regions were extracted from the Harvard-Oxford atlas using FSL (Smith et al. 2004), nonlinearly converted from Montreal Neurological Institute (MNI) coordinates into Talairach coordinates using the MNI2TAL program (http://imaging.mrc-cbu.cam.ac.uk/imaging/MniTalairach) and combined in XBAM. Within the mask, <1 false-positive cluster was expected with thresholds of *P* < 0.05 for voxel and *P* < 0.03 for cluster comparisons.

## Results

### Participant Characteristics

Groups did not differ in age or IQ (Table 1). As expected, groups differed on SDQ total and sub-scores. Post hoc tests correcting for multiple comparisons showed that all groups differed on SDQ total-scores (all Log(BF_10_) > 3, *P* < 0.001). ASD boys were more impaired on peer, prosocial and hyperactivity/inattention subscales compared to typically developing controls and OCD boys (all Log(BF_10_) > 4, *P* < 0.001), who did not differ. On the conduct sub-scale, ASD boys differed from controls only (Log(BF_10_) = 2.64, *P* < 0.003). On the emotion sub-scale, controls differed from ASD and OCD boys (both Log(BF_10_) > 7, *P* < 0.001), who did not differ from each other.

**Table 1:** Participant characteristics

Variables	TDC (*N* = 20) Mean (SD)	ASD (*N* = 24) Mean (SD)	OCD (*N* = 20) Mean (SD)	*F*-test (DF)	*P*-value	Log (BF_10_)
Age (years)	15.1 (2.0)	14.6 (1.6)	15.7 (1.4)	2.7 (2,61)	0.08	−0.03
IQ	119.7 (11.9)	113.1 (14.3)	117.7 (13.4)	1.4 (2,61)	0.25	−0.99
SCQ total score (*t*-test)	2.2 (2.3)	16.5 (7.4)	—	8.3 (42)	<0.001	17.26
SDQ total score	5.0 (3.9)	19.5 (6.8)	12.5 (5.6)	36.2 (2,61)	<0.001	19.03
SDQ emotional distress	0.7 (1.7)	4.3 (2.8)	4.4 (2.6)	14.6 (2,61)	<0.001	7.88
SDQ conduct	0.9 (1.3)	2.6 (2.2)	1.9 (1.5)	5.6 (2,61)	0.006	2.07
SDQ peer relations	1.6 (2.5)	6.5 (2.4)	3.3 (3.0)	19.8 (2,61)	<0.001	11.05
SDQ hyperactive impulsive/inattentive	2.2 (1.9)	6.2 (2.4)	3.0 (2.7)	17.9 (2,61)	<0.001	9.96
SDQ prosocial behavior	8.6 (2.4)	4.5 (2.4)	7.7 (2.6)	17.4 (2,61)	<0.001	9.68
ADOS communication score	—	3.6 (1.2)	—	—	—	
ADOS social interaction score	—	9.0 (2.3)	—	—	—	
ADOS communication+social	—	12.7 (3.1)	—	—	—	
ADOS stereotypy score	—	1.5 (1.5)	—	—	—	
ADI communication score	—	16.6 (4.7)	—	—	—	
ADI social interaction score	—	20.0 (5.3)	—	—	—	
ADI repetitive behavior score	—	6.5 (2.4)	—	—	—	
CY-BOCS total score	—	—	22.3 (5.8)	—	—	
CY-BOCS–obsessions	—	—	10.8 (3.6)	—	—	
CY-BOCS–compulsions	—	—	12.0 (3.1)	—	—	

Abbreviations: ADI, Autism Diagnostic Interview; ADOS, Autism Diagnostic Observation Schedule; ASD, Autism Spectrum Disorder; CY-BOCS, Childrens’ Yale-Brown Obsessive-Compulsive Symptom Checklist; DF, degrees of freedom; SD, standard deviation; SDQ, Strengths and Difficulties Questionnaire. TDC, typically developing controls. Note, Log(BF_10_) is reported for Bayesian analyses, as BF_10_ values were consistently high.

### Performance Data

Groups did not differ on their preference ratio for safe decks across the entire task (BF_10_ = 0.16, *F*_(2,63)_ = 0.65, *P* = 0.53) or in group-by-block (4 blocks of 20 trials each) interaction analysis (BF_10_ = 0.01, *F*_(2,62)_ = 0.35, *P* = 0.71), with strong evidence in favor of the null hypothesis (BF_01_ = 219.05). Task performance is further summarized in Supplementary Figures S1 and S2.

### Movement

Groups did not significantly differ on minimum (BF_10_ = 0.13, *F*_(2,63)_ = 0.03, *P* = 0.97), maximum (BF_10_ = 0.36, *F*_(2,63)_ = 1.37, *P* = 0.26) or mean (BF_10_ = 0.19, *F*_(2,63)_ = 0.49, *P* = 0.61) head-translation in 3D-Euclidian space.

### Model Comparison

We first tested which model provided the best fit for the data by comparing WAIC scores (Supplementary Table S1), with lower WAIC scores indicating better model-fits. Results suggested that the VPP model (WAIC_total_ = 11387.78) provided the best model-fit relative to the other 2 models (PVL-DecayRI WAIC_total_ = 12502.34; PVL-Delta WAIC_total_ = 12812.60) in all 3 groups, consistent with previous studies (Worthy, Pang et al. 2013; Ahn et al. 2014).

We used the winning VPP model to compare parameter estimates among groups (Table 2). Typically developing controls showed greater choice sensitivity (*c*) compared to ASD (95% HDI from 0.83 to 4.54, mean of HDI = 2.69; *t*(20.4) = 32.93, *P* < 0.001) and OCD boys (95% HDI from 1.44 to 4.22, mean of HDI = 2.83; *t*(19.2) = 34.19, *P* < 0.001). Controls also showed higher reinforcement learning weights (ω) than ASD (95% HDI from 0.46 to 0.98, mean of HDI = 0.72; *t*(23.6) = 26.13, *P* < 0.001) and OCD boys (95% HDI from 0.45 to 0.97, mean of HDI = 0.71; *t*(20.2) = 39.96, *P* < 0.001). ASD boys showed greater perseverance decay rates (*k*) compared to controls (95% HDI from −0.44 to −0.06, mean of HDI = −0.25; *t*(33.8) = −5.21, *P* < 0.001) and OCD boys (95% HDI from 0.005 to 0.47, mean of HDI = 0.24; *t*(42) = 3.75, *P* = 0.001). A complete table of differential distributions is presented in Supplementary Table S2.

**Table 2:** Parameter estimates from the VPP model

Parameter	TDC (*N* = 20) Mean (SD)	ASD (*N* = 24) Mean (SD)	OCD (*N* = 20) Mean (SD)
Learning rate (*A*)	0.01 (0.01)	0.44 (0.22)	0.24 (0.15)
Feedback sensitivity (α)	0.14 (0.06)	0.61 (0.13)	0.96 (0.43)
Choice sensitivity (*c*)	3.16 (0.33)	0.72 (0.07)	0.66 (0.02)
Loss aversion (λ)	0.22 (0.08)	4.70 (1.65)	4.91 (2.27)
Loss impact (ε_p_)	−1.38 (0.87)	−1.69 (2.97)	−1.80 (1.16)
Gain impact (ε_n_)	−0.84 (1.33)	−0.76 (2.75)	−1.07 (2.16)
Perseverance decay rate (*k*)	0.42 (0.08)	0.63 (0.17)	0.44 (0.16)
Reinforcement learning weight (ω)	0.94 (0.01)	0.25 (0.13)	0.26 (0.08)

Abbreviations: ASD, Autism Spectrum Disorder; SD, standard deviation; TDC, typically developing controls; VPP, value-plus-perseverance.

### Group Maps of Brain Activation

Images of within-group brain activation for choice (risky vs. safe), anticipation, and outcome (win vs. loss) phases are presented in Supplementary Figure S3.

### Group Effect-Choice

**Figure 2.**
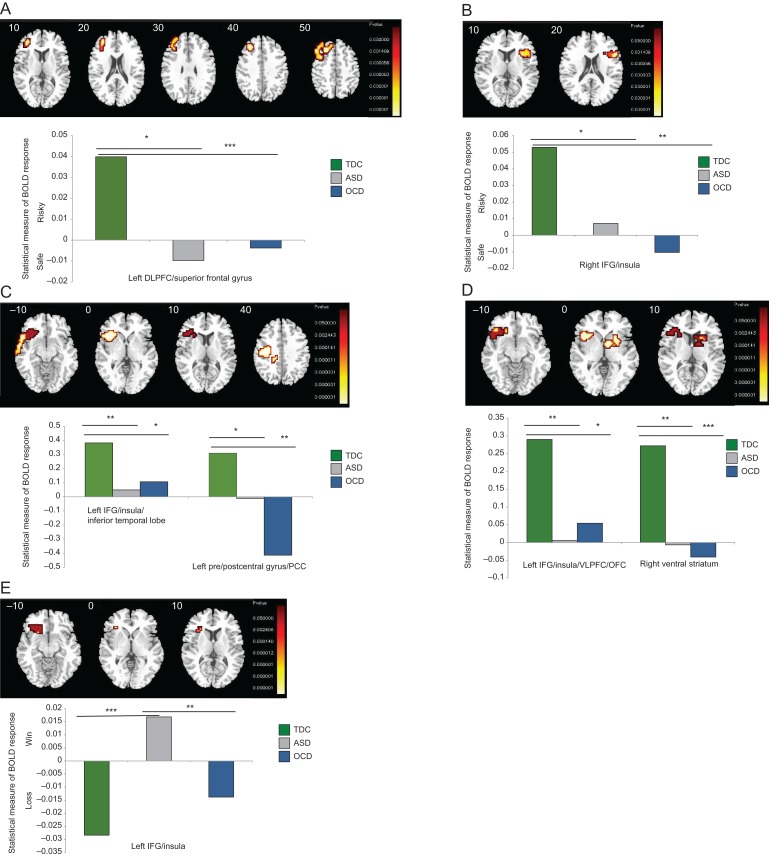
Between-group differences in brain activation between control boys, boys with autism spectrum disorder (ASD) and boys with OCD. Analysis of variance (ANOVA) showing the main effect of group on brain activation for the 3 phases of the IGT. (*A*) Whole-brain results of the group effect during decision-making (choice phase, safe vs. risky), (*B*) Region of interest (ROI) results of the group effect during decision-making (choice phase, safe vs. risky), (*C*) Whole-brain results of the group effect during outcome anticipation, (*D*) ROI results of the group effect during outcome anticipation, (*E*) ROI results of the group effect during outcome presentation (win vs. loss). Talairach z-coordinates are shown for slice distance (in mm) from the intercommissural line. The right side of the image corresponds with the right side of the brain. * indicates significance at the *P* < 0.05 level, ** indicates significance at the *P* < 0.01 level, *** indicates significance at the *P* < 0.001 level.

Whole-brain analysis of covariance (ANCOVA) including age as a covariate compared brain activation during the choice phase (risky vs. safe choices) and showed a main effect of group in left DLPFC extending into superior frontal gyrus (Table 3**A**; Fig. 2*A*). Post hoc comparisons revealed that this was due to controls activating this region more during risky choices relative to both ASD (BF_10_ = 82.98, *P* < 0.001) and OCD subjects (BF_10_ = 13.97, *P* = 0.02).

**Table 3:** ANCOVA results of brain activation differences between typically developing control boys, boys with ASD, and boys with OCD

Contrast	Regions of activation	Brodmann areas	Peak Talairach coordinates (*x*,* y*, *z*)	Voxels	Cluster *P*-value
(A) Choice (risky-safe)					
Whole-brain					
TDC > ASD,OCD	**L DLPFC**, superior frontal gyrus	6/8/9/46	−33,4,64	302	0.004
ROI					
ASD,OCD > TDC	**R IFG**, insula	45	36,22,4	51	0.009
(B) Anticipation (vs. baseline)					
Whole-brain					
TDC > ASD,OCD	**L IFG**, insula, inferior temporal	47	−40,26,−7	198	0.01
TDC > ASD,OCD	**L pre/**postcentral, PCC	6	−36,−15,26	225	0.01
ROI					
TDC > ASD,OCD	**L IFG**, insula, VLPFC, OFC	47	−40,26,−13	83	0.006
TDC > ASD,OCD	**R VS**, NAcc, caudate, putamen	–	7,4,−7	58	0.01
(C) Outcome (win-loss)					
Whole-brain					
No suprathreshold clusters					
ROI					
ASD > C,OCD	**L IFG**/insula	45/47	−33,30,−13	39	0.02

Abbreviations: ASD, Autism Spectrum Disorder; IFG, inferior frontal gyrus; L, left; OFC, orbitofrontal cortex; PCC, posterior cingulate cortex; ROI, region of interest; TDC, typically developing controls; VLPFC, ventrolateral prefrontal cortex; VS, ventral striatum. **BOLD** regions = cluster-peak.

When the search space was constrained to the fronto-striatal ROIs, controls had increased activation to risky choices relative to ASD (BF_10_ = 2.83, *P* = 0.03) and OCD (BF_10_ = 7.89, *P* = 0.005) boys in right IFG/insula (Table 3A; Fig. 2*B*). No group differences were observed in any of the other ROIs.

Excluding the 4 medicated OCD boys from analyses had no effect on the main findings.

### Group Effect-Anticipation

Whole-brain ANCOVA comparing brain activation during outcome anticipation showed a group-effect in 2 regions: left IFG/insula/inferior temporal lobe and left pre/postcentral gyrus extending into PCC. This was due to shared underactivation in both regions in ASD (left IFG/insula/inferior temporal lobe: BF_10_ = 164.47,* P* = 0.003; pre/postcentral gyrus/PCC: BF_10_ = 5.25, *P* = 0.05) and OCD boys (left IFG/insula/inferior temporal lobe: BF_10_ = 8.29,* P* = 0.04; pre/postcentral gyrus/PCC: BF_10_ = 55.60,* P* = 0.002) relative to typically developing controls (Table 3B; Fig. 2*C*).

ROI analysis revealed 2 clusters that significantly differed among groups, one of which was observed in the whole-brain analysis (see above): left IFG/insula, extending in the ROI analysis into VLPFC/OFC, and in right ventral striatum (VS), including nucleus accumbens, caudate and putamen. Post hoc comparisons showed shared reduction in both clusters in ASD (IFG/insula/OFC: BF_10_ = 79.65, *P* = 0.002; VS: BF_10_ = 101.61,* P* = 0.004) and OCD (IFG/insula/OFC: BF_10_ = 7.82,* P* = 0.04; VS: BF_10_ = 122.07,* P* < 0.001) boys versus typically developing controls (Table 3B; Fig. 2*D*).

When the 4 medicated OCD boys were excluded from analyses, all group-difference clusters remained, but the difference in the right VS cluster from the ROI analysis was observed only at a reduced threshold of *P* = 0.07 in patients relative to controls.

### Group Effect-Outcome

Whole-brain analyses comparing activation differences during outcome presentation showed no effect of group when wins versus losses were contrasted. However, ROI analysis revealed a group effect in the left IFG/insula, which was due to ASD boys having disorder-specific enhanced activation to wins relative to typically developing controls (BF_10_ = 237.61, *P* < 0.001) and OCD boys (BF_10_ = 31.60, *P* = 0.003), who had more activation in this region to losses relative to ASD boys (Table 3**C**; Fig. 2*E*). Excluding the 4 medicated OCD boys had no effect on the main findings. Moreover, when all analyses were repeated excluding age as a covariate, results remained largely unchanged.

### Associations Between Symptom Measures and Task Performance/Brain Activation

After correction for multiple comparisons, there was no relationship between symptom measures and any parameter estimate or overall advantageous preference ratio in the ASD or OCD group. There was no statistically significant correlation between symptom measures and brain activation among ASD or OCD boys.

### Associations Between Task Performance and Brain Activation

In the control group, higher advantageous preference ratios were associated with increased activation to risky versus safe choices in left DLFPC (*r* = 0.43, BF_10_ = 7.99, *P* = 0.007), and with increased activation during outcome anticipation in left IFG (*r* = 0.45, BF_10_ = 11.12, *P* = 0.005).

Parameter estimates or overall performance were not associated with brain activation in ASD or OCD boys.

## Discussion

This is the first study to investigate the underlying neural correlates of IGT performance both in ASD and OCD and the first study to compare the 2 disorders in fMRI during decision-making. Individuals with ASD and OCD shared differences in decision-making strategies with regard to decreased choice consistency and reliance on reinforcement learning compared to controls, in order to achieve overall similar task performance compared to typically developing boys. Furthermore, ASD and OCD boys showed shared neurofunctional underactivation relative to controls during decision-making in left dorsolateral prefrontal and right inferior fronto-insular regions and in lateral inferior/orbitofronto-striatal regions and PCC during outcome anticipation. During outcome presentation, however, ROI analyses showed that ASD boys had disorder-specific enhanced activation to wins versus losses in a left inferior fronto-insular region relative to OCD boys and controls.

The computational modeling results suggest that, despite overall comparable performance to typically developing controls, ASD and OCD boys used shared decision-making strategies that differed from controls to achieve this performance. OCD and ASD participants were less consistent in their choices, in line with previous evidence of increased switching behavior on the IGT in ASD adolescents (Johnson et al. 2006; Yechiam et al. 2010) that may relate to underlying difficulties with implicit learning and cognitive flexibility (Johnson et al. 2006; Solomon et al. 2015). The present work extends this evidence to OCD, suggesting that increased exploration (independent of outcome sensitivity) may be a shared trans-diagnostic behavioral phenotype of decision-making. Moreover, the finding of lower reinforcement learning weights in both patient groups compared to typically developing controls suggests that ASD and OCD individuals less effectively implemented reversal-learning strategies to maximize outcomes and instead used a different strategy (e.g., exploration), in line with impaired reward learning in OCD (Nielen et al. 2009) and ASD (Scott-Van Zeeland et al. 2010). Taken together, this suggests that patients may achieve performance similar to controls via enhanced exploration and less reliance on learning from experienced outcomes. Moreover, perseverance strengths decayed at a faster rate in the ASD group compared to the OCD and control groups, in line with evidence that ASD individuals have a tendency to switch decks more frequently (Johnson et al. 2006). This effect may be dissociable from the disorder-shared decreased choice consistency that was also observed in OCD, as choices on previous decks have less influence on future choices, regardless of reward/punishment valuation on a given deck.

Whole-brain fMRI analysis results showed that both patient groups shared reduced activation in left DLPFC during decision-making relative to typically developing controls, and these results were extended to the right IFG/insula in ROI analyses. Lateral PFC is important for value representation (Ridderinkhof et al. 2004), and more specifically, DLPFC has been implicated in working memory, important for incorporating known information during decision deliberation (Li et al. 2010). DLPFC activation during decision-making under ambiguity has consistently been observed in typically developing populations (Krain et al. 2006). Moreover, ventrolateral prefrontal regions and the insula are related to emotional attribute of decision options and are part of a proposed “saliency network” implicated in stimulus significance and affective response (Phillips et al. 2003). IGT performance and neural representation of decision values in dorso- and ventrolateral PFC mature with age, suggesting development of a decision-making network incorporating action values with executive processes (Christakou, Gershman et al. 2013). Thus, the present findings could imply abnormalities in the functional maturation of these regions in ASD and OCD. Furthermore, enhanced activation in left DLPFC to risky versus safe decks was related to better performance in controls, whereas this relationship was not observed in ASD or OCD individuals. Given the DLPFC's role in integrating memory representations with goal-directed behavior (Ridderinkhof et al. 2004), this may suggest that ASD and OCD individuals have neurofunctional deficits in updating reward expectation. Moreover, in ASD, reduced DLPFC activation has been found during reversal-learning, suggesting that abnormalities in this region may relate to problems in flexibly updating choice behavior due to abnormalities with implicit learning that may also influence choice consistency on the IGT (D'Cruz et al. 2016).

Whole-brain results showed that both patient groups relative to typically developing controls had reduced activation in left OFC/VLPFC/IFG/insula during outcome anticipation. These results were confirmed as well as extended to right BG/VS in ROI analyses. This is in line with evidence in OCD of decreased lateral orbitofrontal activation during outcome presentation on a reversal-learning task (Remijnse et al. 2006; Chamberlain et al. 2008) and reward anticipation (Jung et al. 2011) and extends this evidence to ASD. In OCD, OFC deficits have been linked to impaired reward-related learning and to an inability to detect changes in reinforcement contingencies (Menzies et al. 2008), and the present findings suggest that this phenotype may be shared with ASD, in line with evidence in ASD of fronto-limbic abnormalities during reward gain/loss, independent of valence (Kohls et al. 2013). Moreover, cognitive inflexibility has been associated with OCD, affecting goal-directed decision-making and learning (Gillan and Robbins 2014). A previous study found that OCD adolescents had reduced left IFG activation compared to controls during set-shifting (Britton et al. 2010). Moreover, a study of reward reversal-learning found that ASD adults had reduced VS as well as left DLFPC and parietal activation compared to controls (D'Cruz et al. 2016), in line with our findings of disorder-shared reduced activation in these regions, implicating these areas in a range of reward-related processes that may be affected in both ASD and OCD.

The basal ganglia, and more specifically the caudate and VS, have been consistently implicated in reward expectation and value representation (Dichter, Damiano et al. 2012). This region is particularly relevant to OCD given the prominence of fronto-striatal networks in the neurofunctional characterization of the disorder (Menzies et al. 2008). ROI findings of disorder-shared blunted VS response during reward anticipation are in line with previous findings of similar underactive VS response during ambiguous reward anticipation in ASD (Dichter, Felder et al. 2012; Kohls et al. 2013; D'Cruz et al. 2016) and OCD (Menzies et al. 2008; Figee et al. 2011) as well as depression (Smoski et al. 2009) and schizophrenia (Juckel et al. 2006), suggesting the possibility of a shared neurobiology among a range of disorders with regard to fronto-striatal under-responsiveness to anticipated reward.

ROI analyses revealed that ASD boys had disorder-specific increased activation in left IFG/insula to positive (wins) versus negative (losses) feedback relative to OCD boys and typically developing controls, who both had more activation to loss in this region. Some studies have found insula hyperactivation during reward in ASD (Cascio et al. 2012; Dichter, Richey et al. 2012), and another found enhanced left frontal activation in ASD individuals during rewarded outcomes (Schmitz et al. 2008), implying that reward-related left frontal systems are enhanced in ASD (Cascio et al. 2012). This is in line with the insula's role in interoceptive awareness as part of the proposed “saliency network” (Critchley et al. 2004; Menon and Uddin 2010), suggested to be affected in ASD individuals (Uddin and Menon 2009), and suggests that similar systems are intact in OCD patients during reward processing.

This study has several limitations. While psychiatric comorbidity was an exclusion criterion, we cannot discard the possibility that subthreshold symptoms of other disorders were present in our sample. Moreover, ASD participants were not assessed using OCD-specific measures, e.g., CY-BOCS, (and vice-versa). Nonetheless, thorough clinical assessment of ASD and OCD participants and inclusion of mostly medication-naïve patients are study strengths, and absence of comorbidity was confirmed by a consultant psychiatrist in all cases. Four OCD boys were prescribed SSRIs. Although there is evidence for neurofunctional effects of serotonin during decision-making (Murphy et al. 2008), results largely remained when medication was accounted for, although the right VS cluster was seen only at a reduced threshold, suggesting a possibility that medication may have influenced brain activation during reward anticipation in this region. However, it is more likely that this secondary analysis was underpowered. Moreover, we found no association between symptom severity and performance measures, which is possibly due to patient/symptom heterogeneity in our clinical groups. However, it is also possible that, while fMRI analyses were adequately powered to detect neurobiological differences (Thirion et al. 2007), correlation analyses may have been underpowered to detect behavioral associations, and behavioral analyses may have been underpowered to detect effects on the somewhat simplistic measure of advantageous preference ratio. Future studies should aim to also assess trans-diagnostic, trait-based measures that may more accurately capture individual differences or cognitive/behavioral subtypes within each disorder.

The aim of this study was to compare as a first step relatively “pure” cases of disorders to understand disorder-specific abnormalities. However, given the common co-occurrence between ASD and OCD, future studies should investigate to what extent the comorbid presentation of ASD and OCD differs from the pure disorders to elucidate the underlying neural mechanisms underlying this overlap and co-occurrence. Understanding the neurobiology of the comorbid condition and whether related neural dysfunction resembles brain dysfunction typical of ASD or of OCD independently would also be very relevant for treatment. In line with a recent study comparing these groups during temporal discounting (Carlisi et al. 2017), another “hot” EF task, there were predominantly shared neurofunctional abnormalities between ASD and OCD. However, another recent study comparing these groups during sustained attention, a “cool” EF task, found predominantly OCD-specific abnormalities that were not observed in ASD boys compared to controls (Carlisi, Norman, Murphy et al. 2016). The main aim of comparing different diagnostic groups with fMRI is to identify shared and different underlying neurobiological substrates that could be targeted in interventions (e.g., pharmacological, behavioral, neurofeedback). If we are able to understand the fine-grained cognitive and neurofunctional mechanisms driving differences and similarities between ASD and OCD patients and typically developing adolescents, and if the findings are replicated across future studies and across a wider range of tasks, this could potentially have implications for findings of disorder-specific biomarkers that could be targeted in differential treatments for the 2 disorders. Therefore, the present findings suggest that diagnostic differentiation may not map on to neurobiological differentiation in the context of “hot” EF and that treatments could exploit the neurofunctional abnormalities that are shared in these disorders. For example, brain stimulation or fMRI neurofeedback studies could target regions such as the dmPFC that are involved in “hot” EF and implicated in both disorders. In line with this, it is interesting to note that SSRIs are often used in the treatment of individuals with ASD and with OCD (Soomro et al. 2008; Benvenuto et al. 2013), providing further support for shared biological mechanisms underpinning specific aspects of these disorders that may have treatment implications. However, such theories should be empirically tested, and these 2 different diagnostic groups should be compared to a comorbid group to elucidate the underlying neurofunctional substrates of the co-occurring presentation that would be important for the development of neuroscience-based treatment for psychiatric disorders.

## Conclusions

This first behavioral and fMRI comparison of ASD and OCD adolescents on the IGT showed that ASD and OCD patients used different decision-making strategies relative to typically developing controls in that they were less consistent in their choices and relied less on reinforcement learning to achieve overall performance comparable to controls. ASD adolescents, moreover, had distinctive perseverative task performance in that they showed higher perseverance decay rates compared to OCD and typically developing boys. This was underpinned by predominantly shared neurofunctional deficits relative to typically developing controls in dorsal and ventral prefrontal regions during decision-making and in orbitofrontal-ventral striatal regions during reward and loss processing, as shown by both whole-brain and ROI analyses. ASD patients, however, had disorder-specific enhanced inferior frontal/insular activation to reward feedback in the ROI analysis, suggesting a possible neurofunctional signature of reward-based decision-making on the IGT that may be unique to ASD. This study provides novel insight into underlying neurobiological and behavioral mechanisms that shed light on trans-diagnostic phenotypes of reward learning and decision-making in the 2 disorders that may drive respective clinical characteristics of executive impairments in each disorder.

## Supplementary Material

IGT_Supplement_R1_Final
