# Supplementary material for: Shared and Disorder-Specific Neurocomputational Mechanisms of Decision-Making in Autism Spectrum Disorder and Obsessive-Compulsive Disorder
Source: Cereb Cortex. 2017 Oct 17;27(12):5804–16. doi: 10.1093/cercor/bhx265 (PMC6919268; doi:10.1093/cercor/bhx265)
Supplement: IGT_Supplement_R1_Final [file igt_supplement_r1_final.pdf]

Shared and disorder-specific neurocomputational mechanisms of decision-making in Autism Spectrum Disorder and Obsessive-Compulsive Disorder – Supplementary Information

Christina O Carlisi, Luke Norman, Clodagh M. Murphy, Anastasia Christakou, Kaylita Chantiluke, Vincent Giampietro, Andrew Simmons, Michael Brammer, Declan G. Murphy, MRC AIMS consortium, David Mataix-Cols, Katya Rubia

Methods

Participants

All but 3 ASD participants scored above clinical threshold for ASD on the Social Communication Questionnaire (SCQ (Rutter et al., 2003)), but these patients were included on the basis of clinician-confirmed ASD diagnosis. Six ASD participants also scored above threshold for inattention/hyperactivity symptoms on the Strengths and Difficulties Questionnaire (SDQ (Goodman and Scott, 1999)) but were not excluded on the basis that attention problems are common in ASD and clinician confirmation that ASD symptoms were the sole/primary clinical concern for these patients.

One OCD patient scored above clinical cut-off for inattention/hyperactivity symptoms on the SDQ but was not excluded on the basis that communication and attention difficulties can be misconstrued for OCD-related symptoms and the fact that no OCD patients met criteria for ASD or ADHD based on clinical interview.

OCD patient medication status

Patient 1: Sertraline 75mg

Patient 2: Sertraline 100mg

Patient 3: Sertraline 200mg

Patient 4: Fluvoxamine 100mg; risperidone 0.5mg

PVL-DecayRI and PVL-Delta models

In the prospect valence learning models, outcome evaluation is assessed according to the prospect utility function. The utility $u(t)$ on trial t of each outcome $x(t)$ is expressed as:

$$u(t) = \begin{matrix} x(t)^{a} & if\ x(t) \geq 0 \\ -\lambda|x(t)|^{a} & if\ x(t) < 0 \end{matrix} \qquad (1)$$

α (0< α<2) determines the shape of the utility function, and the loss-aversion parameter λ (0< λ <10) determines sensitivity to losses versus gains. Higher α implies greater sensitivity to feedback, and a value of λ<1 indicates higher sensitivity to gains than losses (whereas λ>1 indicates the opposite).

The PVL models are identical except that they use different learning rules. The parameter *A* determines how much past expectancy is discounted; in the decayRI learning rule, expectancies of all decks are discounted on each trial, and the expectancy of the chosen deck is updated by current outcome utility:

$$E_j(t+1) = A \cdot E_j(t) + \delta_j(t) \cdot u(t) \qquad (2)$$

In the delta rule, only the expectancy of the selected deck is updated while expectancies of the other decks is unchanged:

$$E_j(t+1) = E_j(t) + A \cdot \delta_j(t) \cdot (u(t) - E_j(t)) \qquad (3)$$

Thus, learning rate *A* determines how much weight is placed on past experiences vs. most recent experience of the chosen deck. A high learning rate indicates that the recent outcome has a large influence on expectancy of the chosen deck (i.e. ‘forgetting’ is more rapid) while a low learning rate indicates the opposite. Next, a softmax function (Luce, 1959)

is used to calculate the probability of choosing deck j, with sensitivity (θ) determining the degree of exploitation vs. exploration. *c* is a choice consistency (sensitivity) parameter:

$$\Pr[D(t+1)=j]=\frac{e^{\theta\cdot E_j(t+1)}}{\sum_{k=1}^{4}e^{\theta\cdot E_k(t+1)}} \qquad (4)$$

Value-Plus-Perseverance model

Evidence suggests that participants frequently use a win-stay-lose-switch (WSLS) strategy, that is, a perseverative strategy that cares only about the last choice's outcome for making a choice on the current trial during reward-based learning and decision-making (Worthy et al., 2013). Based on a model comparison between the *PVL-DecayRI* and WSLS models showing that each model respectively was the best fit for only half of the subjects investigated, a hybrid VPP model was developed (Worthy et al., 2013) combining the PVL-Delta and perseverance heuristic. This model assumes that individuals track expectancies ($E_j(t)$) and perseverance strengths ($P_j(t)$); expectancies are computed using the learning rule of the PVL-Delta model, and three additional perseverance parameters are included:

$$P_j(t+1)=\begin{matrix} k\cdot P_j(t)+\varepsilon_p & if\ x(t)\geq 0 \\ k\cdot P_j(t)+\varepsilon_n & if\ x(t)<0 \end{matrix} \qquad (5)$$

k ($0<k<1$) determines how much perseverance strengths of all (including unselected) decks decay on each trial, and ε_p and ε_n indicate loss/gain impact, respectively, on choice behaviour. Positive values reflect a tendency to persevere on the same deck, while negative values indicate a tendency to switch decks on the next trial. Overall value, $V_j(t+1)$ is the weighted sum of $E_j(t+1)$ and $P_j(t+1)$:

$$V_j(t+1)=\omega\cdot E_j(t+1)+(1-\omega)\cdot P_j(t+1) \qquad (6)$$

ω is the reinforcement learning (RL) weight (0<ω<1); a low ω indicates the subject relies less on RL/more on perseverance. Choice probability was again computed using the softmax function, but with $V_j(t+1)$:

$$\Pr[D(t+1)=j]=\frac{e^{\theta \cdot V_j(t+1)}}{\sum_{k=1}^{4} e^{\theta \cdot V_k(t+1)}} \qquad (7)$$

Hierarchical Bayesian Analysis

HBA is a more suitable approach for parameter estimation compared to e.g. Maximum Likelihood Estimation (MLE) for considering individual differences through the use of posterior distributions and Markov chain Monte Carlo (MCMC) sampling algorithms (Ahn et al., 2014). Parameter estimates obtained through traditional methods such as MLE are generally estimated at the individual level from point estimates that maximize the likelihood of data for each individual subject (Myung, 2003). However, these MLE estimates can be noisy, particularly in samples with insufficient amounts of data. To address this, group-level analysis estimating a single set of parameters for an entire group may provide more reliable estimates but consequently ignores fine-grained individual differences (Ahn et al., 2016).

Bayesian statistics rely on the use of prior distributions, estimating model parameters and updating these prior using posterior distributions on a trial-by-trial basis given the data using Bayes' rule. In HBA, hyper-parameters are derived in addition to parameters introduced at the individual level (Gelman et al., 2014). These hyper-parameters are set with group-level means and standard deviations, where the resulting joint posterior distribution $P(\Theta, \Phi|D)$ is defined as:

$$P(\Theta, \Phi|D)=\frac{P(D|\Theta, \Phi)\mathrm{P}(\Theta,\Phi)}{P(D)} \propto P(D|\Theta)P(\Theta|\Phi)P(\Phi) \qquad (8)$$

This hierarchical structure of HBA leads to a “shrinkage effect”, i.e. individual estimates are pulled closer to the group mean because they inform the group’s estimate, which in turn informs the estimates of each individual (Gelman et al., 2014). As a result, parameter estimates of each individual tend to be more stable and less noisy, as common factors among individuals are informed by group tendencies. This HBA approach is particularly beneficial when e.g. the number of trials is too small to precisely estimate individual parameters for each subject, as is likely the case in the 80-trial version of the IGT used in this study. Such advantages have been demonstrated by simulation studies showing that HBA outperforms MLE in parameter recovery (Ahn et al., 2011). Lastly, HBA provides full posterior distributions rather than point estimates, thus facilitating group comparisons in a Bayesian manner (Guitart-Masip et al., 2012). For further information on HBA and its implementation in hBayesDM, refer to (Ahn et al., 2016).

fMRI data analysis methods

Individual-level analysis

Data were first processed to minimize motion-related artefacts (Bullmore et al., 1999a). A 3D volume consisting of the average intensity at each voxel over the entire experiment was calculated and used as a template. The 3D image volume at each time point was then realigned to this template by computing the combination of rotations (around the *x*, *y* and *z* axes) and translations (in *x*, *y* and *z* dimensions) that maximised the correlation between the image intensities and the volume in question and the template (rigid-body registration). Following realignment, data were then smoothed using a Gaussian filter (full-width at half-maximum (FWHM) 7.2 mm) to improve the signal-to-noise ratio of the images (Bullmore et al., 1999a). Following motion correction, global detrending and spin-excitation history correction, time series analysis for each subject was conducted based on a previously

published wavelet-based resampling method for fMRI data (Bullmore et al., 1999b, Bullmore et al., 2001). At the individual subject level, a standard general linear modelling approach was used to obtain estimates of the response size (beta) to each of the task conditions (choice, anticipation and outcome phases) against an implicit baseline. We first convolved the main experimental conditions with 2 Poisson model functions (peaking at 4 and 8s). We then calculated the weighted sum of these 2 convolutions that gave the best fit (least-squares) to the time series at each voxel. A goodness-of-fit statistic (SSQ ratio) was then computed at each voxel consisting of the ration of the sum of squares of deviations from the mean intensity value due to the model (fitted time series) divided by that of the squares due to the residuals (original time series minus model time series). The appropriate null distribution for assessing significance of any given SSQ ratio was established using a wavelet-based data re-sampling method (Bullmore et al., 2001) and applying the model-fitting process to the resampled data. This process was repeated 20 times at each voxel, and the data was combined over all voxels, resulting in 20 null parametric maps of SSQ ratios for each subject. These maps were then combined to give the overall null distribution of SSQ ratio. This same permutation strategy was applied at each voxel to preserve spatial correlation structure in the data. Individual SSQ ratio maps were then transformed into standard space, first by rigid-body transformation of the fMRI data into a high-resolution inversion recovery image of the same subject, and then by affine transformation onto a Talairach template (Talairach and Tournoux, 1988).

Group-level analysis

For the group-level analysis, less than 1 false positive-activated 3D cluster was expected at $p<0.05$ (voxel-level) and $p<0.01$ (cluster-level). A group-level activation map was produced for each group and each experimental condition (choice, anticipation, outcome)

by calculating the median observed SSQ ratios at each voxel in standard space across all subjects and testing them against the null distribution of median SSQ ratios computed from the identically transformed wavelet-resampled data (Brammer et al., 1997, Bullmore et al., 2001). The voxel-level threshold was first set to 0.05, and tests were conducted to identify voxels that might be plausibly activated followed by a test at a cluster-level threshold of $p<0.01$ to remove the false-positive clusters produced by the voxel-level test (Bullmore et al., 1999b, Bullmore et al., 2001). Next, a cluster-level threshold was computed for the resulting 3D voxel clusters. The necessary combination of voxel and cluster-level thresholds was not assumed from theory but rather was determined by direct permutation for each dataset, giving excellent type-II error control (Bullmore et al., 1999b). Cluster mass rather than a cluster extent threshold was used to minimize discrimination against possible small, strongly responding foci of activation (Bullmore et al., 1999b).

Results

Task performance

Supplementary Figure S1. Overall advantageous preference ratio

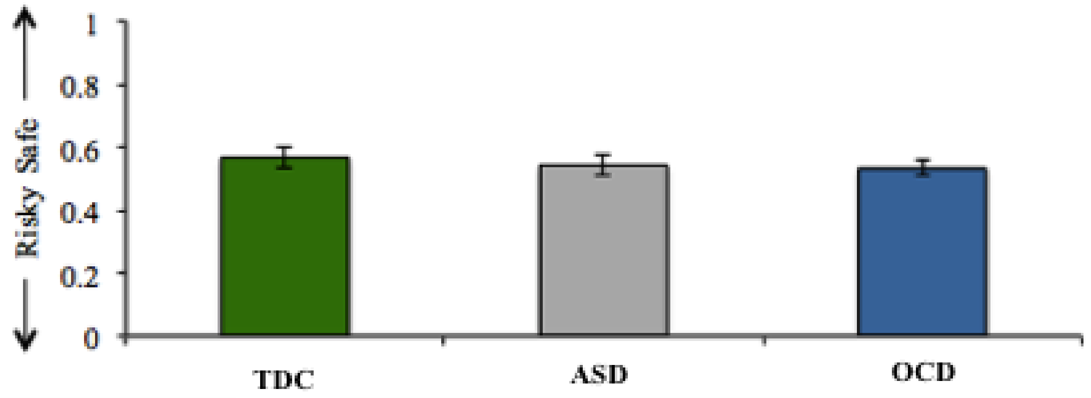

Supplementary Figure S2. Advantageous preference ratio split by task-block

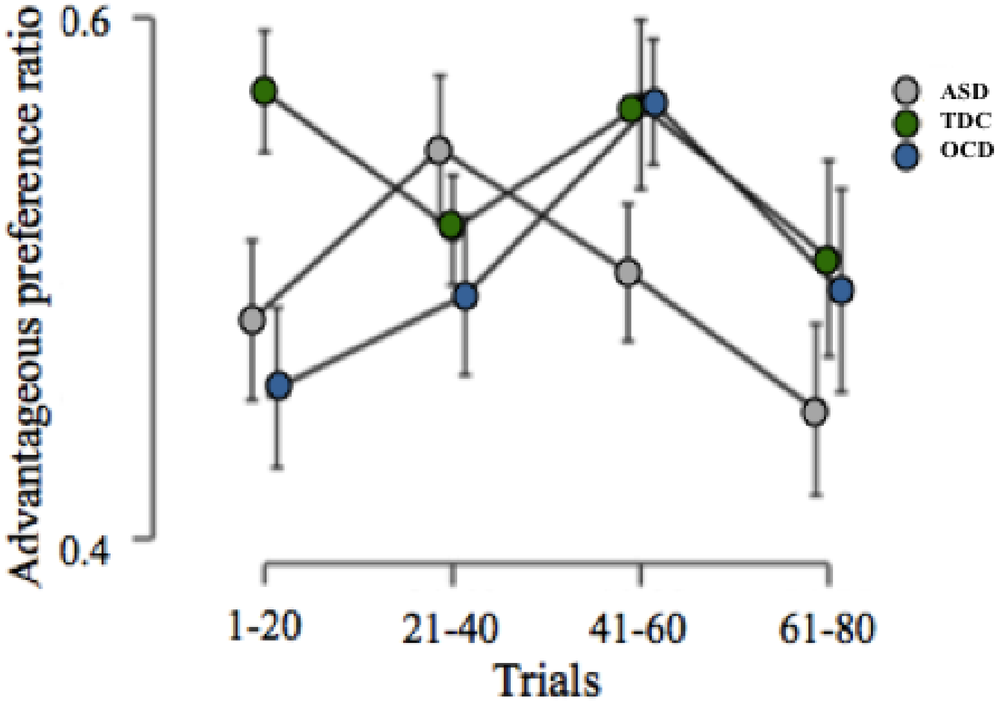

Model comparison – WAIC scores

Supplementary Table S1. WAIC scores for each model

Model	$WAIC_{TDC}$	$WAIC_{ASD}$	$WAIC_{OCD}$	$WAIC_{SUM}$
PVL-DecayRI	4025	4502	3975	12502
PVL-Delta	4075	4676	4061	12813
VPP	3616	4130	3642	11388

Abbreviations: ASD, Autism Spectrum Disorder; OCD, Obsessive-Compulsive Disorder; PVL, Prospect Valence Learning; RI, Reinforcement Learning; TDC, Typically Developing Controls; VPP, Value Plus Perseverance

Differential distributions of VPP model parameters

Supplementary Table S2. Highest Density Intervals for two-way comparisons

**Parameter**	**95% HDI of MCMC**	
	**TDC vs. ASD**	
A	-0.922	0.004
α	-1.775	0.181
c	0.832	4.539
λ	-9.377	0.031
ε_p	-2.105	1.329
ε_n	-1.663	1.289
k	-0.442	-0.061
ω	0.461	0.981
	**TDC vs. OCD**	
A	-0.494	0.016
α	-1.923	0.031
c	1.436	4.217
λ	-9.328	0.025
ε_p	-1.278	1.850
ε_n	-1.249	1.516
k	-0.220	0.211
ω	0.455	0.974
	**ASD vs. OCD**	
A	-0.282	0.900
α	-1.708	1.064
c	-0.640	1.622
λ	-7.999	7.165
ε_p	-1.135	2.265
ε_n	-1.227	1.995
k	0.005	0.468
ω	-0.394	0.357

Abbreviations: α , outcome sensitivity; *A* , learning rate; ASD, Autism Spectrum Disorder; c, consistency/choice sensitivity; ε_p /ε_n, impact of gain/loss, respectively, on perseverance behaviour; HDI, highest density interval; *k* , perseverance decay rate; λ, loss aversion; MCMC, Markov Chain Monte Carlo sampling; OCD, Obsessive-Compulsive Disorder; TDC, Typically Developing Control; ω, reinforcement learning weight.

Within-group fMRI results

Supplementary Figure S3. Within-group brain activation maps

Within-group maps for choice phase (red: risky>safe; blue: safe>risky)

(A) Typically Developing Control Boys

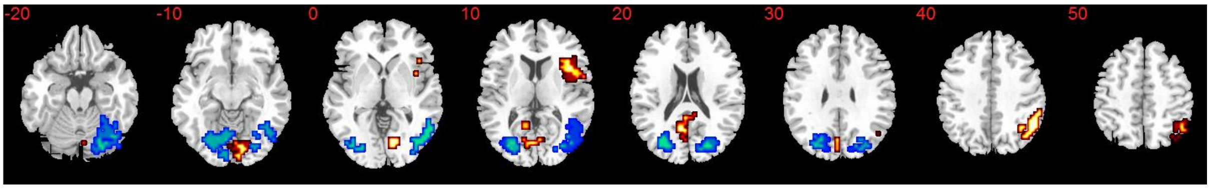

(B) ASD Boys

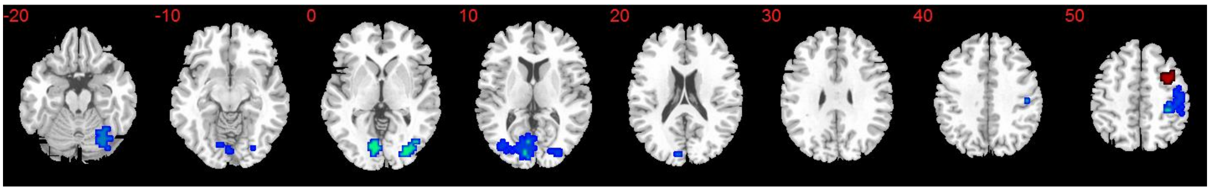

(C) OCD Boys

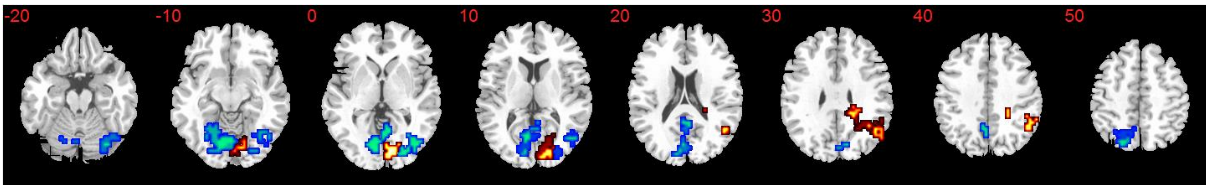

Within-group maps for anticipation phase (red: increased; blue: decreased; anticipation>baseline)

(A) Typically Developing Control Boys

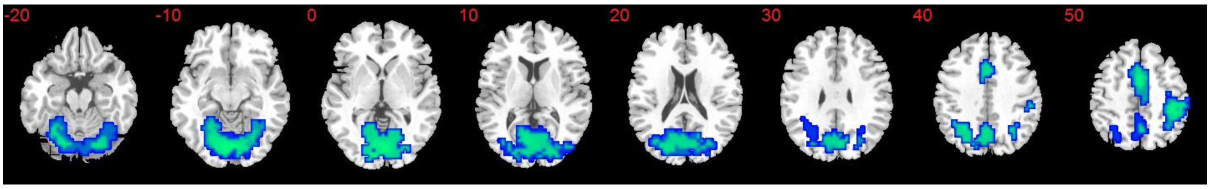

(B) ASD Boys

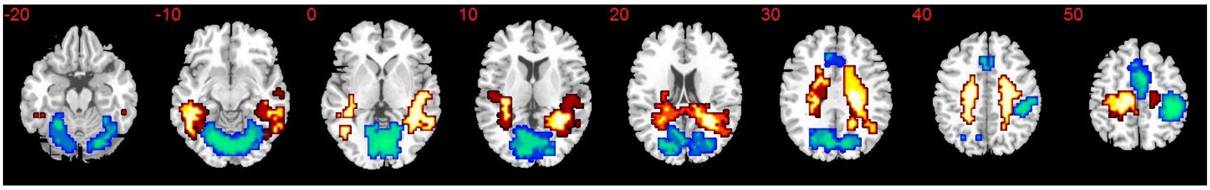

(C) OCD Boys

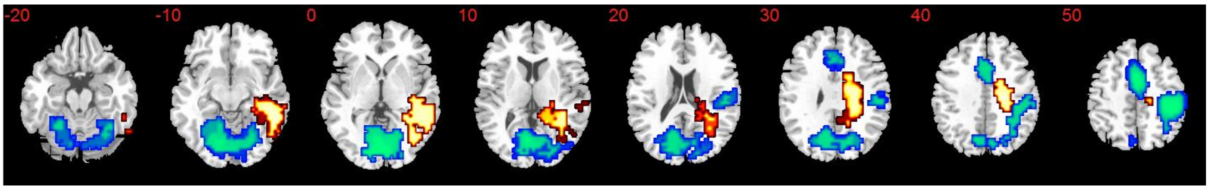

Supplementary Figure S1 (continued). Within-group brain activation maps

Within-group maps for outcome phase (red: win>loss; blue: loss>win)

(A) Typically Developing Control Boys

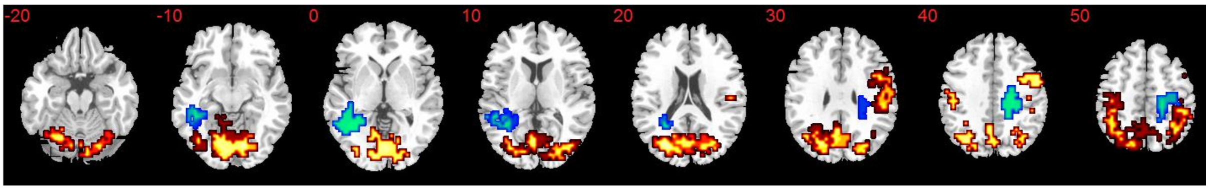

(B) ASD Boys

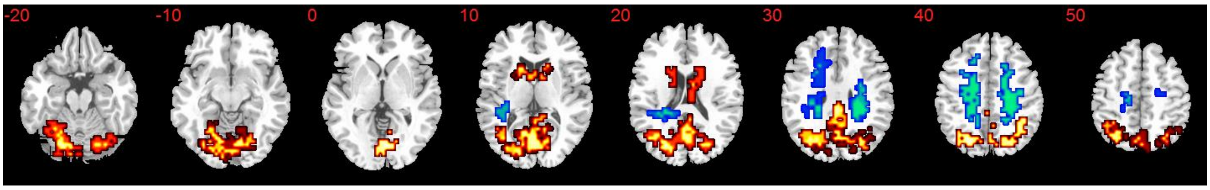

(C) OCD Boys

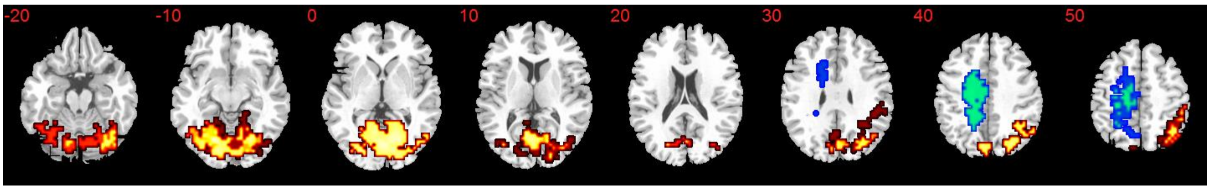

Supplementary Figure S1. Horizontal sections showing within-group brain activation for each task condition (choice, anticipation, outcome) for (A) typically developing control boys, (B) boys with ASD and (C) boys with OCD. Talairach *z*-coordinates are shown for slice distance (in mm) from the intercommisural line. The right side of the image corresponds with the right side of the brain.

References

AHN, W.-Y., HAINES, N. & ZHANG, L. (2016). Revealing neuro-computational mechanisms of reinforcement learning and decision-making with the hBayesDM package. *bioRxiv*.

AHN, W.-Y., KRAWITZ, A., KIM, W., BUSEMEYER, J. R. & BROWN, J. W. (2011). A model-based fMRI analysis with hierarchical Bayesian parameter estimation. *Journal of Neuroscience, Psychology, and Economics*. 4(2): 95.

AHN, W.-Y., VASILEV, G., LEE, S.-H., BUSEMEYER, J. R., KRUSCHKE, J. K., BECHARA, A. & VASSILEVA, J. (2014). Decision-making in stimulant and opiate addicts in protracted abstinence: evidence from computational modeling with pure users. *Frontiers in Psychology*. 5(849).

BRAMMER, M. J., BULLMORE, E. T., SIMMONS, A., WILLIAMS, S. C. R., GRASBY, P. M., HOWARD, R. J., WOODRUFF, P. W. R. & RABE-HESKETH, S. (1997). Generic brain activation mapping in functional magnetic resonance imaging: A nonparametric approach. *Magnetic Resonance Imaging*. 15(7): 763-770.

BULLMORE, E., BRAMMER, M., RABE-HESKETH, S., CURTIS, V., MORRIS, R., WILLIAMS, S., SHARMA, T. & MCGUIRE, P. (1999a). Methods for diagnosis and treatment of stimulus-correlated motion in generic brain activation studies using fMRI. *Human Brain Mapping*. 7(1): 38-48.

BULLMORE, E., LONG, C., SUCKLING, J., FADILI, J., CALVERT, G., ZELAYA, F., CARPENTER, T. A. & BRAMMER, M. (2001). Colored noise and computational inference in neurophysiological (fMRI) time series analysis: resampling methods in time and wavelet domains. *Human Brain Mapping*. 12(2): 61-78.

BULLMORE, E. T., SUCKLING, J., OVERMEYER, S., RABE-HESKETH, S., TAYLOR, E. & BRAMMER, M. J. (1999b). Global, voxel, and cluster tests, by theory and permutation, for a difference between two groups of structural MR images of the brain. *Medical Imaging, IEEE Transactions on Medical Imaging*. 18(1): 32-42.

GELMAN, A., CARLIN, J. B., STERN, H. S. & RUBIN, D. B. (2014). *Bayesian data analysis*. Chapman & Hall/CRC Boca Raton, FL, USA.

GOODMAN, R. & SCOTT, S. (1999). Comparing the Strengths and Difficulties Questionnaire and the Child Behavior Checklist: Is Small Beautiful? *Journal of abnormal child psychology*. 27(1): 17-24.

GUITART-MASIP, M., HUYS, Q. J. M., FUENTEMILLA, L., DAYAN, P., DUZEL, E. & DOLAN, R. J. (2012). Go and no-go learning in reward and punishment: Interactions between affect and effect. *NeuroImage*. 62(1): 154-166.

LUCE, R. D. (1959). *Individual Choice Behavior: A Theoretical Analysis*. Wiley: New York.

MYUNG, I. J. (2003). Tutorial on maximum likelihood estimation. *Journal of Mathematical Psychology*. 47(1): 90-100.

RUTTER, M., BAILEY, A. & LORD, C. (2003). *The social communication questionnaire: Manual*. Western Psychological Services.

TALAIRACH, J. & TOURNOUX, P. (1988). *Coplanar stereotaxic atlas of the human brain, a 3-dimensional proportional system: an approach to cerebral imaging*. Thieme: New York.

WORTHY, D. A., HAWTHORNE, M. J. & OTTO, A. R. (2013). Heterogeneity of strategy use in the Iowa gambling task: A comparison of win-stay/lose-shift and reinforcement learning models. *Psychonomic Bulletin & Review*. 20(2): 364-371.
